# The contribution of staff call light response time to fall and injurious fall rates: an exploratory study in four US hospitals using archived hospital data

**DOI:** 10.1186/1472-6963-12-84

**Published:** 2012-03-31

**Authors:** Huey-Ming Tzeng, Marita G Titler, David L Ronis, Chang-Yi Yin

**Affiliations:** 1 Department of Nursing, The University of Michigan-Flint, School of Health Professions and Studies, 303 E. Kearsley Street, 2180 WS White Building, Flint, MI 48502, USA; 2 School of Nursing, The University of Michigan, Ann Arbor, MI 48109, USA; 3 Veterans Affairs Center for Clinical Management Research, Ann Arbor, MI 48113, USA; 4 Department of History, Chinese Culture University, Taipei, Taiwan

## Abstract

**Background:**

Fall prevention programs for hospitalized patients have had limited success, and the effect of programs on decreasing total falls and fall-related injuries is still inconclusive. This exploratory multi-hospital study examined the unique contribution of call light response time to predicting total fall rates and injurious fall rates in inpatient acute care settings. The conceptual model was based on Donabedian's framework of structure, process, and health-care outcomes. The covariates included the hospital, unit type, total nursing hours per patient-day (HPPDs), percentage of the total nursing HPPDs supplied by registered nurses, percentage of patients aged 65 years or older, average case mix index, percentage of patients with altered mental status, percentage of patients with hearing problems, and call light use rate per patient-day.

**Methods:**

We analyzed data from 28 units from 4 Michigan hospitals, using archived data and chart reviews from January 2004 to May 2009. The patient care unit-month, defined as data aggregated by month for each patient care unit, was the unit of analysis (*N *= 1063). Hierarchical multiple regression analyses were used.

**Results:**

Faster call light response time was associated with lower total fall and injurious fall rates. Units with a higher call light use rate had lower total fall and injurious fall rates. A higher percentage of productive nursing hours provided by registered nurses was associated with lower total fall and injurious fall rates. A higher percentage of patients with altered mental status was associated with a higher total fall rate but not a higher injurious fall rate. Units with a higher percentage of patients aged 65 years or older had lower injurious fall rates.

**Conclusions:**

Faster call light response time appeared to contribute to lower total fall and injurious fall rates, after controlling for the covariates. For practical relevance, hospital and nursing executives should consider strategizing fall and injurious fall prevention efforts by aiming for a decrease in staff response time to call lights. Monitoring call light response time on a regular basis is recommended and could be incorporated into evidence-based practice guidelines for fall prevention.

## Background

The effects of fall prevention programs on decreasing total falls and fall-related injuries are still inconclusive [1-6]. To design a sustainable fall prevention program, objective, staff-centered indicators of fall and injurious fall rates must first be identified through research. Staff response time to call lights, one such indicator, is primarily determined by nurses' reaction to each call light and may be linked to the circumstances present when a call is initiated. Staff response time to call lights has been recognized as an indicator reflecting the reality of patients' hospitalization experiences and provides an overall understanding of the patterns of an inpatient care unit's care delivery [7,8].

Recently discharged older patients have emphasized that nurses should provide assistance and answer a call light in a timely manner [9]. One of the patients' major safety concerns during their hospital stay was lack of availability of nurses to help when needed. In a qualitative study to understand why hospitalized patients fall in acute care hospitals, nurses and assistants expressed that having nursing staff work together to rapidly answer call lights is essential to preventing patient falls [10]. A common assumption is that a quick response by a nurse to a call light paired with fewer unmet patient needs translates to less opportunity for a patient to fall [11-14]. This assumption still needs to be tested empirically.

### Purpose of this study and hypotheses

This exploratory multi-hospital study examined the unique contribution of call light response time to total fall rates and injurious fall rates in adult inpatient care units in hospitals. The covariates included the hospital, unit type, total nursing hours per patient-day (HPPDs), percentage of the total nursing HPPDs supplied by registered nurses (RNs), percentage of patients aged 65 years or older, average case mix index (CMI), percentage of patients with altered mental status, percentage of patients with hearing problems, and call light use rate per patient-day. The patient care unit-month was the unit of analysis. We analyzed data from 28 units from 4 Michigan hospitals using archived hospital data and reports collected between January 2004 and May 2009.

Two research hypotheses were tested: (1) call light response time will contribute significantly to predicting fall rates after controlling for the covariates, and (2) call light response time will contribute significantly to predicting injurious fall rates after controlling for the covariates.

### Conceptual framework

**Figure 1:**
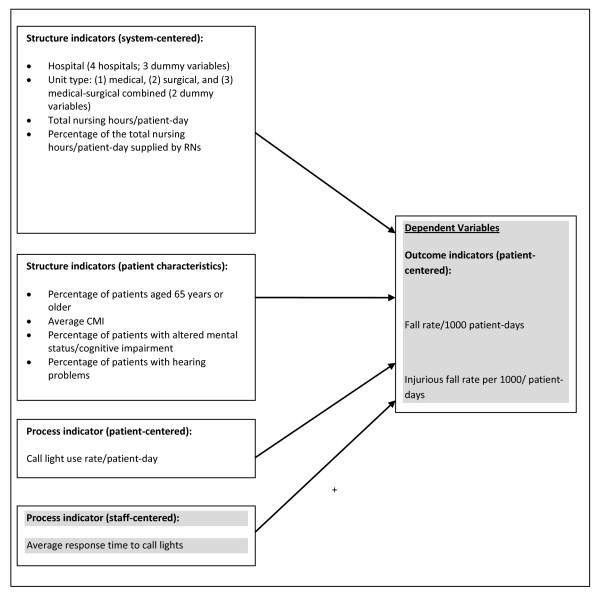
**The conceptual model of inpatient falls with a focus on staff response time to call lights in adult acute inpatient care settings**.

The National Quality Forum (NQF) [15] suggested outcome, process, structure, and patient-centered measures as considerations for supporting internal health-care organization quality improvement. Using this approach to assess falls, we defined the outcome measures to include the total fall rate and injurious fall rate. The conceptual model for this study, depicted in Figure 1, was based on Donabedian's [16,17] framework of structure, process, and health-care outcomes and the NQF approach to fall and injurious fall prevention. As shown in Figure 1, this model was used to examine the relationship between call light response time (1 staff-centered process indicator) and fall rates and injurious fall rates (2 patient-centered outcome indicators), while controlling for the covariates. Covariates included 4 system-centered structural indicators, 4 unit-level patient characteristics, and 1 patient-centered process indicator in adult inpatient acute care units. The total fall rates and injurious fall rates were conceptualized as patient-centered outcome indicators. Call light response time was conceptualized as a staff-centered process indicator because staff members decide when to respond to patients' call lights.

The hospital, unit type (i.e., medical, surgical, and medical-surgical combined units), total nursing hours HPPDs, and percentage of the total nursing HPPDs supplied by RNs were conceptualized as system-centered structure indicators. Based on a previous multi-hospital study, fall characteristics can differ by hospital type [18]. Additionally, a qualitative study in an acute medical unit in Michigan [13,19] found that factors associated with patient room design and settings, hospital equipment, and human resources may contribute to falls. Another study [20] showed that fall rates, related injuries, and circumstances of inpatient falls varied significantly among clinical departments, probably due to differences in patient characteristics. Two previous studies [7,21] conducted in a single hospital concluded that the contribution of the average call light response time to the total fall and injurious fall rates varies across unit types. The potential effects of the total nursing HPPDs on call light response time remained inconclusive [22], but the effects on fall occurrence were supported by previous research [15,23].

For exploratory purposes, 4 unit-level patient characteristics conceptualized as structure indicators were also included as covariates, including percentage of patients aged 65 years or older, average CMI, percentage of patients with altered mental status, and the percentage of patients with hearing problems. The mean CMI at discharge represents the average diagnosis-related group relative weight for that unit-month. Falls in a general hospital were related to advanced age, patients' acuity levels, altered mental status, and sensory deficits [23-25]. The CMI value is used to define the average acuity levels for patients admitted to a hospital [26]. Sensory deficits (hearing problems) and altered mental status may contribute to patient-nurse communication breakdown (e.g., being unable to understand or follow nurses' instructions), which may result in hospital-acquired injury [27]. The potential relationships between the patient characteristics and call light response time have never been studied systematically.

The call light use rate per patient-day was conceptualized as a patient-centered process indicator because patients or their families are the ones who determine whether and when to push the call buttons. Previous studies [7,21,22] showed that the call light use rate was significantly correlated with the average response time to call lights. Consequently, the call light use rate was included as one of the covariates.

## Methods

### Design and settings

This exploratory study was conducted at 4 hospitals in the Midwestern United States and used archived hospital data and reports. Twenty-eight adult medical, surgical, and medical-surgical inpatient acute care units provided the data. Due to the difference across study hospitals in backing up archived data, the covered data periods varied between hospitals.

The study hospitals included the following: Hospital 1, academic medical center, bed size about 900, 14 participating units, data from January 2004 to December 2008; Hospital 2, community hospital, bed size about 300, 4 participating units, data from February 2007 to December 2008; Hospital 3, teaching hospital, bed size about 900, 4 participating units, data from April 2008 to May 2009; and Hospital 4, teaching hospital, bed size about 700, 6 participating units, data from January 2006 to December 2008. Data were included from different time periods to increase the sample size.

The unit of analysis was the patient care unit-month (abbreviated as unit-month) defined as data aggregated by month for each patient care unit. Some interdependence for the data points from a single unit and for the data points from other units in the same hospital existed. For statistical analyses and result interpretation, each data point for a study unit was assumed to be independent from all others. The study was approved by each hospital's institutional review board and the corresponding author's employer university. There was no conflict of interest.

### Data sources and collection

In each study hospital, a designated site coordinator (a hospital staff or administrator) retrieved the archived hospital data and facilitated chart reviews. Each site coordinator was instructed by the corresponding author about the desired hospital data to be used to ensure the consistency and reliability of the data across the 4 study hospitals. Under the corresponding author's supervision, the retrieved data were entered by a trained research assistant and verified by another trained research assistant for accuracy. Detailed information about the study variables are described in Table 1.

**Table 1:** Study variables and definitions

	Data source	Conceptual definition	Operational definition
Dependent variables			
Fall rate	Incident reports/fall incident report database	The fall rate was defined as the rate at which patients fall during hospital stays/1000 patient-days [15]. A fall was defined as an unplanned descent to the floor with or without injury. All falls are included, whether they result from physiologic or environmental causes [28].	(counts of total falls × 1000)/(total patient-days)
Injurious fall rate	Incident reports/fall incident report database	The injurious fall rate was defined as the fall rate/1000 inpatient-days at which physical injury occurs, regardless of severity (including minor/resulted in application of a dressing, moderate, major injury, and death) [15,28].	(counts of injury falls × 1000)/(total patient-days)
Covariates			
Hospital	As identified by each study hospital	Four hospitals served as study sites: one academic medical center (Hospital 1); 2 teaching hospitals (Hospitals 2 and 4); and one community hospital (Hospital 3). This study used 3 dummy variables to capture 4 study hospitals, instead of hospital characteristics (e.g., bed size and teaching status), to control for the variations across study hospitals.	3 dummy variables were included in the regression model. Hospital 1 was used as a reference group; Hospital 2: 1 = Hospital 2, 0 = all other hospitals; Hospital 3: 1 = Hospital 3, 0 = all other hospitals; Hospital 4: 1 = Hospital 4, 0 = all other hospitals. When the values of Hospital 2, Hospital 3, and Hospital 4 = 0, Hospital 1 would be identified.
Unit type	As identified by each study hospital	The unit classification of each study unit, as designated by the hospital, was identified by the designated site coordinate. The unit type included 3 categories: (1) medical, (2) surgical, and (3) medical-surgical combined.	Two dummy variables were included in the regression model. Medical units were used as the reference group (unit type 2: 1 = surgical unit, 0 = all other units; and unit type 3: 1 = medical-surgical combined unit, 0 = all other units). When the values of unit type 2 and unit type 3 = 0, medical units would be identified.
Total nursing hours per patient-day (HPPDs)	The payroll database	As a system-centered measure, this was defined as the number of productive hours worked by nursing staff with direct care responsibilities per patient-day [15].	Total nursing hours/total patient-days
Percentage of the total nursing HPPDs supplied by registered nurses	The payroll database	As a system-centered measure, this was defined as the percentage of the productive nursing HPPDs worked by RNs with direct care responsibilities to the number of total productive nursing HPPDs worked by nursing staff with direct care responsibilities [15].	(Total nursing HPPDs supplied by RNs/total nursing HPPDs) × 100%
Patient age in percentage of patients aged ≥ 65 years	Patient management database	The percentage of all patients discharged from the study unit during the defined time period, who were ≥ 65 years.	(Sum of the years of the discharged patients in age/total discharged patients) × 100%
Case mix index (CMI)	Patient management database	CMI value mean of all patients discharged from the study unit during the defined time period represents the average diagnosis-related group relative weight for that unit-month. The CMI value is used to define the average acuity for patients admitted to a particular hospital [26].	(Sum of the CMI values of the discharged patients/total discharged patients) × 100%
Percentage of patients with altered mental status	Chart review of the nursing notes at admission	The percentage of patients hospitalized at the study unit on the 15th of the first month of each quarter, who had cognitive impairment or altered mental status. The charts of 10 randomly sampled patients per study unit were reviewed. If any cognitive impairment or altered mental status was identified in the chart at admission, this patient was coded as Yes (1); otherwise, No (0) was coded.	(Number of patients with cognitive impairment or altered mental status/10) × 100%
Percentage of patients with hearing problem	Chart review of the nursing notes at admission	The percentage of patients hospitalized at the study unit on the 15th of the first month of each quarter, who had hearing problems. The charts of a total of 10 randomly sampled patients per study unit were reviewed. If any hearing problems (with or without correction) were identified in the chart at admission, this patient was coded as Yes (1); otherwise, No (0) was coded.	(Number of patients with hearing problems/10) × 100%
Call light use rate per patient-day	The reports generated from the call light tracking system adopted by each hospital	Patient/family-initiated calls made from the pillow speaker or call cord attached to the wall in the patient rooms are categorized as normal calls (excluding the calls initiated in the bathrooms). In this project, only normal calls were studied. The normal call count will include all the calls either cancelled at the console or at the stations of origin (i.e., the patient's room).	(Counts of normal calls/number of the covered days) × (total number of days for the mo.)/(total patient-days for the month) Due to the skewed distribution, this continuous variable was recorded into 10 equal groups and labeled in percentiles (10 = least frequent, 100 = most frequent). The recoded variable was analyzed as a continuous variable.
Predictor Average response time to call lights	The reports generated from the call light tracking system adopted by each hospital	Patient/family-initiated calls made from the pillow speaker or call cord attached to the wall in the patient rooms are categorized as normal calls (excluding the calls initiated in the bathrooms). In this project, only normal calls were studied. The response time was defined as the time elapsed from normal call activation to call cancellation from the patient room. The response times were aggregated at the unit level for each month, and calculated by: (call light response time in seconds for all the calls made for the unit and month)/(total number of calls for the unit and month).	The average time for "Staff Response" on the reports generated from the call light tracking system was calculated as: (Sum of the call light response time for the calls in seconds)/(total call light use). Due to the skewed distribution, this continuous variable was recorded into 10 equal groups and labeled in percentiles (10 = fastest, 100 = slowest). The recoded variable was analyzed as a continuous variable.

The 2 dependent variables were the fall rate and the injurious fall rate. The fall rate was defined as the rate at which patients fell during their hospital stays/1000 patient-days [15]. A fall was defined as an unplanned descent to the floor with or without injury. All falls types were included, whether falls resulted from physiologic or environmental causes [28]. The operational definition of the fall rate was (number of total falls × 1000)/(total patient-days). The injurious fall rate was defined as the fall rate/1000 inpatient-days during which physical injury occurred, regardless of severity (including minor, moderate, major injury and death) [15,28]. The operational definition of the injurious fall rate was (number of injury falls × 1000)/(total patient-days).

The predictor was the average response time to call lights. These data were retrieved from the call light tracking system at each hospital. Patient/family-initiated calls made from the pillow speaker or call cord were categorized as normal calls but calls initiated in the bathrooms were not included in the analysis. The response time was defined as the time that elapsed between a normal call activation to its cancellation from the patient room. The response times for "staff response" on the reports generated from the call light tracking system were aggregated at the unit level for each month and calculated by (call light response time in seconds for all the calls made for the unit and month)/(total number of calls for the unit and month). The operational definition of this variable was (sum of the call light response time for the calls in seconds)/(total call light use).

As for covariates, the data on the percentages of patients with altered mental status and hearing problems came from chart review. Due to constrained resources, one data point by quarter for each patient care unit was collected. The percentages of patients hospitalized at the study unit on the 15th of the first month of each quarter, who had cognitive impairment or altered mental status at admission, were calculated. As for the chart review procedure, the charts of 10 randomly sampled patients per study unit were reviewed by a trained research assistant. If altered mental status was identified at admission in the chart, the patient was coded as Yes (1); otherwise, No (0) was coded (Table 1).

For each study hospital, the patient management database was used to generate the total patient-days per unit-month. The daily count of total patient-days was the midnight census. The daily counts for a unit for a specified month were added up to indicate the total patient-days for that unit and month. The designated site coordinators calculated this variable (the total patient-days per unit-month) before sending the data to the corresponding author. Total patient-days per unit-month were used to compute the call light use rate per patient-day and fall and injurious fall rates.

### Data management

Data were entered into the Statistical Package for the Social Sciences (SPSS; 18.0 Window version; SPSS Inc., Chicago, IL, USA). All data points were matched by patient care unit as well as by year and month. Only unit-month data with valid fall rate and injurious fall rate data were included in the analysis.

In the course of data management, means and standard deviations were calculated for the continuous variables, and the skewness and kurtosis values of these variables were examined. The call light use rate per patient-day (skewness value = 11.57; kurtosis value = 235.82) and the call light response time (skewness value = 11.00; kurtosis value = 137.61) had high skewness and kurtosis values. The log transformation was done on both variables, but the log transformation left the distributions still skewed. As a result, the continuous variable of the patient call light use rate per patient-day was recorded to fall within 1 of 10 groups: 10 = low to 0.95; 20 = more than 0.95 and up to 3.52; 30 = more than 3.52 and up to 4.66; 40 = more than 4.66 and up to 5.83; 50 = more than 5.83 and up to 6.91; 60 = more than 6.91 and up to 7.56; 70 = more than 7.56 and up to 8.12; 80 = more than 8.12 and up to 8.65; 90 = more than 8.65 and up to 9.65; and 100 = more than 9.65. In addition, the continuous variable of the call light response time (in seconds) was recorded to fall within 1 of 10 groups: 10 = low to 128.10; 20 = more than 128.10 and up to 153.00; 30 = more than 153.00 and up to 167.00; 40 = more than 167.00 and up to 179.40; 50 = more than 179.40 and up to 193.00; 60 = more than 193.00 and up to 207.00; 70 = more than 207.00 and up to 221.00; 80 = more than 221.00 and up to 241.00; 90 = more than 8241.00 and up to 730.40; and 100 = more than 730.40. These 2 recoded variables were used to test the hypotheses.

### Data analyses

SPSS was also used for data analyses. The sample (1063 unit-months) was the total number of months with available data for each patient care unit. One-way analysis of variance (ANOVA) tests were conducted to elucidate differences in the study variable means across the 4 study hospitals and 3 unit types. Separate hierarchical multiple regression analyses were used to test the 2 hypotheses. Hierarchical regression is also called sequential regression; predictors are entered into the equation in the order specified by the researcher. Predictors are entered in steps or blocks with each predictor or a set of predictors being assess in terms of what it/they add(s) to the prediction of the dependent variable, after the previous variables have been controlled for [29].

Missing values for the covariates and predictor were replaced by mean values because data were missing at random. Before entering the categorical covariates into the regression models, 2 sets of dummy variables were created to capture 4 hospitals and 3 unit types. Collinearity among the predictor and covariates was a possible concern and was checked [29]; we found that it is not to be a problem. All predictors were included in the analyses.

The covariates were entered into the multiple regression equation first. Then, the average call light response time was entered as a predictor into each model. Key outcomes of the analyses were the significance tests and estimates of regression coefficients for the average call light response time in the final regression models. Alpha was set at 0.05 for the analyses.

The power analysis was used to compute the required sample size. For the power analysis for the multiple linear regression analysis, making the assumption of including up to 13 predictor variables to explain a medium-sized squared multiple correlation (R^2 ^= 0.13) with alpha of 0.05 (2-tailed) and desired statistical power of 0.80 requires a sample size of 149. The total sample size of 1063 unit-months provided more than 99% power; that is, it was more than sufficient. Thus, power was fully adequate for the proposed project [30,31].

## Results

### Descriptive analyses

Table 2 provides descriptive information on study variables for all data points and by hospital. The average total fall rate per 1000 patient-days was 4.08 (SD = 3.06) and the injurious fall rate per 1000 patient-days was 0.91 (SD = 1.11). The average call light response time was 13 minutes and 18 seconds; Hospital 1 had the longest average call light response time (mean, 17 minutes and 27 seconds) and the other 3 hospitals had comparable average response times within the range of 3 minutes and 7 seconds and 3 minutes and 10 seconds.

**Table 2:** Descriptive information of study variables by hospitals and results of one-way between-group analysis of variance (ANOVA) tests on differences of means across 4 hospitals

	**Hospital**^**a**^					
**Variable\Mean (SD)**	**All** **(n = 1063)**	**1** **(n = 750)**	**2** **(n = 92)**	**3** **(n = 56)**	**4** **(n = 165)**	**One-way ANOVA tests (*p*)**

Total fall rate/1000 patient-days	4.08 (3.06)	4.29 (3.24)	3.87 (2.13)	2.96 (1.96)	3.60 (2.78)	F = 5.23** (*p *= 0.001)

Injurious fall rate/1000 patient-days	.91 (1.11)	.97 (1.15)	.36 (.68)	.76 (.71)	1.01 (1.17)	F = 9.22** (*p *< 0.001)

Total productive nursing hours/patient-day	9.23 (2.23)	10.02 (2.09)	5.17 (.99)	9.05 (.73)	8.30 (.66)	F = 217.19** (*p *< 0.001)

Percentage of productive nursing hours provided by RNs	72.90% (8.95)	76.82% (6.82)	58.37% (2.83)	58.87% (2.85)	68.05% (4.36)	F = 413.00** (*p *< 0.001)

Percentage of patients aged ≥ 65 years	35.93% (16.40)	30.13% (13.53)	66.58% (9.18)	47.70% (7.75)	38.10% (8.28)	F = 263.99** (*p *< 0.001)

Average CMI value	1.76 (.72)	1.95 (.78)	1.30 (.24)	1.52 (.35)	1.31 (.26)	F = 59.36** (*p *< 0.001)

Percentage of patients with altered mental status at admission	9.93% (11.91)	7.18% (8.71)	25.11% (16.14)	21.61% (19.33)	8.39% (7.16)	F = 106.94** (*p *< 0.001)

Percentage of patients with hearing difficulties at admission	11.33% (10.96)	13.08% (11.19)	10.87% (10.96)	8.57% (9.42)	5.16% (7.59)	F = 24.93** (*p *< 0.001)

Call light use rate per patient-day	6.43 (5.32)	6.83 (6.03)	4.17 (2.02)	4.78 (2.85)	6.40 (2.55)	F = 8.72** (*p *< 0.001)

Patient call light use rate/patient-day (in 10 equal groups; 10 = least frequent, 100 = most frequent)	54.85 (28.30)	58.20 (30.00)	33.80 (14.96)	40.71 (18.08)	56.12 (21.74)	F = 27.09** (*p *< 0.001)

Call light response time in sec as well as in min and sec	798.34/ 13 min 18 sec (3909.11)	1047.30/17 min 27 sec (4604.87)	190.40/ 3 min 10 sec (61.35)	186.92/3 min 7 sec (30.11)	167.59/ 3 min 8 sec (55.49)	F = 3.50* (*p *= 0.02)

Call light response time (in 10 equal groups; 10 = fastest, 100 = slowest)	54.74 (28.60)	58.54 (28.76)	50.65 (27.53)	48.93 (18.46)	41.16 (27.80)	F = 18.26** (*p *< 0.001)

^a ^Values are means (SD)

*p < 0.05; **p < 0.01

The one-way ANOVA tests were conducted to explore the differences of the study variables across the 4 hospitals and unit types. The analyses showed that there were significant differences in the study variable means across study hospitals (Table 2). The one-way ANOVA tests also showed that only the means of the percentage of productive nursing hours provided by RNs and the staff call light response times were not significantly different across unit types (Table 3).

**Table 3:** Descriptive information of study variables by unit types and results of one-way between-group analysis of variance (ANOVA) tests on differences of means across 3 unit types

	**Unit type**^**a**^			
**Variable\Mean (SD)**	**Medical** **(n = 531)**	**Surgical** **(n = 166)**	**Med-surgical combined** **(n = 366)**	**One-way ANOVA tests (*p*)**

Total fall rate/1000 patient-days	4.52 (3.14)	3.26 (2.73)	3.82 (2.97)	F = 13.14** (*p *< 0 .001)

Injurious fall rate/1000 patient-days	1.03 (1.13)	.74 (1.04)	.81 (1.09)	F = 6.54** (*p *= 0.002)

Total productive nursing hours/patient-day	9.59 (1.69)	8.12 (1.47)	9.27 (3.05)	F = 26.62** (*p *< 0 .001)

Percentage of productive nursing hours provided by RNs	72.87% (6.89)	71.80% (12.49)	73.45% (9.61)	F = 1.94 (*p *= 0.14)

Percentage of patients aged ≥ 65 years	39.09% (18.84)	35.90% (10.35)	31.28% (13.64)	F = 22.98** (*p *< 0 .001)

Average CMI value	1.87 (.84)	1.88 (.46)	1.52 (.56)	F = 26.15** (*p *< 0 .001)

Percentage of patients with altered mental status at admission	10.19% (11.26)	7.53% (8.42)	10.77% (14.02)	F = 4.32* (*p *= 0.01)

Percentage of patients with hearing difficulties at admission	12.53% (11.76)	8.92% (10.45)	10.83% (9.75)	F = 7.29** (*p *= 0.001)

Call light use rate/patient-day	6.00 (3.11)	5.85 (2.70)	7.29 (7.98)	F = 7.39** (*p *= 0.001)

Patient call light use rate/patient-day (in 10 equal groups; 10 = least frequent, 100 = most frequent)	53.20 (28.85)	51.02 (22.53)	58.96 (29.41)	F = 6.34** (*p *= 0.002)

Call light response time in sec as well as in min and sec	599.33/ 9 min 59 sec (3359.26)	548.26/ 9 min 8 sec (1385.52)	1196.54/ 19 min 57 sec (5183.10)	F = 2.86 (*p *= 0.06)

Call light response time (in 10 equal groups; 10 = fastest, 100 = slowest)	54.24 (28.54)	52.55 (29.16)	56.44 (29.68)	F = 1.19 (*p *= 0.30)

^a ^Values are means (SD)

*P < .05; **P < .01

### Testing research hypotheses

Hierarchical multiple regression was used to test the first research hypothesis and to assess how well call light response time could predict the total fall rate per 1000 patient-days, after controlling for the covariates. All covariates explained 8% of the variance in the total fall rate per 1000 patient-days. After entry of the call light response time, the total variance explained by the final model as a whole was 10%, F_13,1049 _= 8.81, *p *< 0.001. The call light response time explained an additional 2% of the variance in the total fall rate (Table 4).

**Table 4:** Summary of results of the final hierarchical multiple regression model: the dependent variable is the total fall rate per 1000 patient-days

	**R**^**2**^	**Adjusted R**^**2**^	F change	Significance
**Initial model summary (covariates only)**	0.08	0.07	7.36	*p *< 0.001**

**Final model summary (covariates and predictor)**	0.10	0.09	24.24	*p *< 0.001**

***Final model***				

ANOVA	**Sum of squares (df)**	**Mean square**	**F value**	**Significance**

Regression	975.75 (13)	75.06	8.81	*p *< 0.001**
Residual	8939.23 (1049)	8.52		
Total	9914.98 (1062)			

**Covariates and predictor**	**Standardized coefficient (β)**	**t**	**Significance**	

(Constant)	--	8.06	*p *< 0.001**	

Hospital 1 (the reference group)	--	--	--	

Hospital 2 (1 = Yes, 0 = No)	-0.11	-2.06	0.04*	

Hospital 3 (1 = Yes, 0 = No)	-0.22	-5.60	*p *< 0.001**	

Hospital 4 (1 = Yes, 0 = No)	-0.05	-1.30	0.19	

Medical unit (the reference group)	--	--	--	

Surgical unit (1 = Yes, 0 = No)	-0.12	-3.16	0.002**	

Medical-surgical combined unit (1 = Yes, 0 = No)	-0.09	-2.60	0.010*	

Total productive nursing hours/patient-day	0.13	3.05	0.002**	

Percentage of productive nursing hours provided by RNs	-0.30	-6.21	*p *< 0.001**	

Percentage of patients aged ≥ 65 years	-0.07	-1.64	0.10	

Average CMI value	0.06	1.75	0.08	

Percentage of patients with altered mental status at admission	0.10	2.77	0.006**	

Percentage of patients with hearing difficulties at admission	0.01	0.26	0.80	

Call light use rate/patient-day (in 10 equal groups; 10 = least frequent, 100 = most frequent)	-0.07	-2.12	0.03*	

Call light response time (in 10 equal groups; 10 = fastest, 100 = slowest)	0.15	4.92	*p *< 0.001**	

*p < 0.05; **p < 0.01

Hierarchical multiple regression was also used to test the second research hypothesis and to assess the ability of the call light response time to predict the injurious fall rate, after controlling for the covariates. All covariates explained 7% of the variance in the injurious fall rate per 1000 patient-days. After entry of the call light response time, the total variance explained by the final model as a whole was 8%, F_13,1049 _= 6.99, *p *< 0.001. The call light response time explained an additional 1% of the variance in the injurious fall rate (Table 5).

**Table 5:** Summary of results of the final hierarchical multiple regression model: the dependent variable is the injurious fall rate per 1000 patient-days

	**R**^**2**^	**Adjusted R**^**2**^	F change	Significance
**Initial model summary (covariates only)**	0.07	0.06	6.97	*p *< 0.001**

**Final model summary (covariates and predictor)**	0.08	0.07	6.78	0.009**

***Final model***				

ANOVA	**Sum of squares (df)**	**Mean square**	**F value**	**Significance**

Regression	104.50 (13)	8.04	6.99	*p *< 0.001**
Residual	1206.50 (1049)	1.15		
Total	1310.99 (1062)			

**Covariates and predictor**	**Standardized coefficient (β)**	**t**	**Significance**	

(Constant)	--	5.17	*p *< 0.001**	

Hospital 1 (the reference group)	--	--	--	

Hospital 2 (1 = Yes, 0 = No)	-0.11	-2.01	0.045*	

Hospital 3 (1 = Yes, 0 = No)	-0.10	-2.37	0.02*	

Hospital 4 (1 = Yes, 0 = No)	0.08	1.79	0.07	

Medical unit (the reference group)	--	--	--	

Surgical unit (1 = Yes, 0 = No)	-0.11	-2.91	0.004**	

Medical-surgical combined unit (1 = Yes, 0 = No)	-0.09	-2.69	0.007**	

Total productive nursing hours/patient-day	0.14	3.12	0.002**	

Percentage of productive nursing hours provided by RNs	-0.20	-4.09	*p *< 0.001**	

Percentage of patients aged ≥ 65 years	-0.13	-3.19	0.001**	

Average CMI value	0.07	1.85	0.07	

Percentage of patients with altered mental status at admission	0.05	1.48	0.14	

Percentage of patients with hearing difficulties at admission	0.01	0.39	0.70	

Call light use rate per patient-day (in 10 equal groups; 10 = least frequent, 100 = most frequent)	-0.07	-2.08	0.04*	

Call light response time (in 10 equal groups; 10 = fastest, 100 = slowest)	0.08	2.60	0.009**	

*p < 0.05; **p < 0.01

## Discussion

### Hypothesis testing

**Figure 2:**
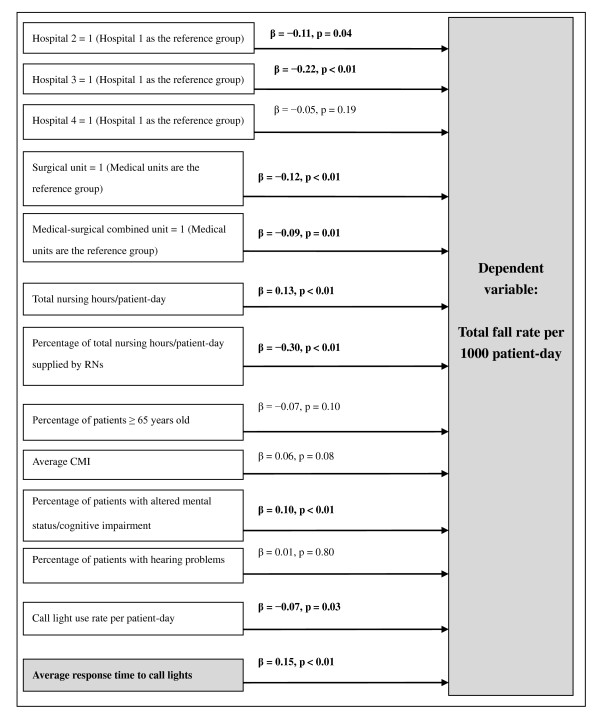
**The tested model of the total fall rate/1000 patient-days with a focus on staff response time to call lights in adult inpatient acute care settings**. The β values with a significance value < 0.05 are highlighted in bold.

The first research hypothesis was supported; that is, shorter call light response time was associated with lower total fall rates. Hospitals 2 and 3 had lower total fall rates compared with Hospital 1. Among units, surgical units and medical-surgical combined units had lower total fall rates than medical units. Fewer total productive nursing hours per patient-day, a higher percentage of productive nursing hours provided by RNs, a lower percentage of patients with altered mental status at admission, a higher call light use rate per patient-day, and faster call light response time would likely lead to a lower total fall rate (Table 4 and Figure 2).

**Figure 3:**
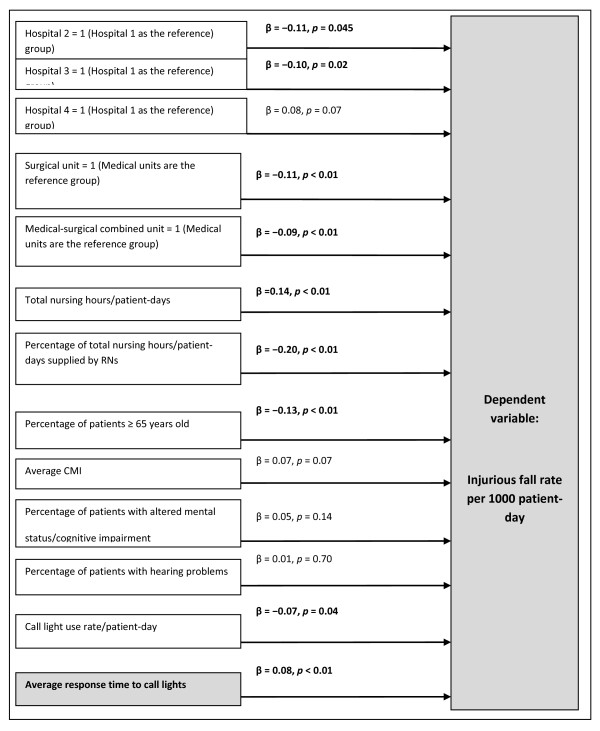
**The tested model of the injurious fall rate/1000 patient-day with a focus on staff response time to call lights in adult inpatient acute care settings**. The β values with a significance value < 0.05 are highlighted in bold.

The second hypothesis was also supported; that is, faster call light response time appeared to contribute to lower injurious fall rates. Hospitals 2 and 3 had lower injurious fall rates compared with Hospital 1. Surgical units and medical-surgical combined units had lower injurious fall rates than medical units. Lower total productive nursing hours per patient-day, a higher percentage of productive nursing hours provided by RNs, a higher percentage of patients aged 65 years or older, a greater call light use rate, and faster call light response time could be expected to contribute to a lower injurious fall rate (Table 5 and Figure 3).

Statistically significant differences were found for both total fall and injurious fall rates across hospitals and unit types. Therefore, fall and injurious fall prevention regimens need to be tailored to different hospitals and unit types, and increasing the percentage of productive nursing hours provided by RNs could be an effective strategy to lower total fall and injurious fall rates.

As for the findings related to patient characteristics and call light use rate, a lower percentage of patients with altered mental status was associated with a lower total fall rate but not a lower injurious fall rate. Units with a higher call light use rate had lower total fall and injurious fall rates. It is arguable that a greater call light use rate and a lower percentage of patients with altered mental status could be integrally linked because it is those with altered mental status who are least likely to activate the call light system for assistance and are most likely to come to harm. It is possible that the method used in this study for identifying those with cognitive impairment or altered mental status (the percentages of patients with altered mental status at admission) inevitably underestimated the impaired patient population. In addition, units with a higher percentage of patients aged 65 years or older had a lower injurious fall rate, but age was not generally correlated with injurious fall rates. To prevent falls and fall-related injuries, regimens should not be determined by patient age.

Overall, the findings of this study were consistent with previous studies [7,21] that more calls for assistance lead to fewer fall-related injuries. The predicting direction of the staff-centered process indicator, call light response time, was also consistent with the one proposed in Figure 1 and the assumption that answering call lights rapidly is essential to prevent patients from falling [10]. In other words, faster call light response time seems to contribute to lower total fall and injurious fall rates. It is recognized that call light response time contributed very little to the overall variance in both of the regression models. This is compounded by the fact that the additional covariates still only contributed to a small amount of the variance, thus highlighting the complexity of factors contributing to patient falls in inpatient acute care settings.

### Study limitations and future research directions

Considering the degrees of freedom for the tested models and the complexity of the results, hierarchical multiple regression analyses were used and linear mixed model analyses were not performed as a study limitation. Without accounting for the clustering within hospitals and within units over time as well as within quarters (two covariates were quarterly data), it is unlikely to have an effect on the estimated effects. However, the tested hierarchical multiple regression models would have standard error terms that are too small and significance tests would be overly sensitive. Also, the findings in this study that are highly significant would be less significant if analyzed correctly (using linear mixed model analyses) and the significant findings might well be non-significant.

Future research may include unmeasured covariates, such as measurements of staff's accountability for performance and patients' values and preferences related to fall prevention interventions. Accountability for performance may include, as an example, the percentage of patients screened for falls and percentage of patients educated about fall prevention strategies and risks [15]. Future research may compare the call light use patterns of hospitalized patients with and without altered mental status (e.g., those with and without diagnoses of delirium and dementia), and link this information to patients' values and preferences related to fall prevention interventions.

## Conclusions

The main finding of this study was that faster call light response time is associated with lower total fall and injurious fall rates, after controlling for the proposed covariates. Call light response time could be a marker of other organizational characteristics and issues that are not easily measured (e.g., nurses' skills, attitudes, behaviors, and an organization's safety and collaboration cultures) which might be associated with falls. In addition, units with a higher percentage of productive nursing hours provided by RNs tended to have lower total fall and injurious fall rates. These results suggested that skill mix is more important than total nursing hours. Hospital and nursing executives should consider strategizing fall and injurious fall prevention efforts by aiming to decrease response time to call lights. Monitoring call light response time on a regular basis is recommended and could be incorporated into evidence-based practice guidelines for fall prevention.

## Competing interests

The authors declare that they have no competing interests.

## Authors' contributions

HMT designed the study design; collected, analyzed, and interpreted the data; and prepared the manuscript. MGT designed the study design and prepared the manuscript. DR designed the study, performed data analysis and interpretation, and prepared the manuscript. CYC performed data analysis and interpretation. All authors read and approved the final manuscript.

## Pre-publication history

The pre-publication history for this paper can be accessed here:

http://www.biomedcentral.com/1472-6963/12/84/prepub
